# Measurement of the normal mandible in neonates in east China

**DOI:** 10.3389/fped.2023.1172909

**Published:** 2023-08-14

**Authors:** Shupei Jiang, Tao Han, Weimin Shen

**Affiliations:** Department of Burns and Plastic Surgery, Children's Hospital of Nanjing Medical University, Nanjing, China

**Keywords:** CT, mandible, 3D reconstruction, measurement, Pierre Robin sequence, Treacher Collins syndrome

## Abstract

**Objective:**

We aimed to measure the related indicators of the neonatal mandible in East China. This provides basic data for the study of the mandible position and morphology of normal newborns and can also provide data support for the diagnosis, evaluation, and treatment of the Pierre Robin sequence.

**Methods:**

First, we collected the CT data of normal neonates at the Nanjing Children's Hospital Affiliated with Nanjing Medical University between January 2013 and January 2019. The data included the maxilla and mandible, and neonates had no craniomaxillofacial-related malformation. We exported the data in DICOM format. In the second step, we imported the data into MIMICS 21.0 to reconstruct the data into a 3D model, and then we used the model to measure the different measurement items. Specific measurement items were as follows: ① Measurement of the angle α: We imported the CT data of the neonate into the software and reconstructed a 3D model. We observed the 3D model to find the left and right gonions (LGo and RGo) and the Menton (Me) and used the angle measurement tool of the software to appoint Me as the apex, and we connected the points LGo, Me, and RGo as angle α. ② Measurement of the distance between the left and right gonions: The distance measurement tool of the software was used to measure the distance between the bilateral gonions as a. ③ Measurement of the distance from the Me to the line between LGo and RGo: The LGo and RGo were connected as a line on the 3D model, then the distance between Me and the line was measured as b. ④ Measurement of the distance between the upper and lower jaw: The median sagittal view was found and the distance c between the foremost point of the upper jaw and the foremost point of the lower jaw was measured. We imported the measurement results into the SPSS software for statistical analysis.

**Results:**

Specific measurement results: ① Angle α: 86.34 ± 8.58°. ② Distance a: 63.63 ± 6.83 mm. ③ Distance b: 31.99 ± 3.70 mm. ④ Distance c: 2.28 ± 1.04 mm. Among all the above indicators, there was no statistical difference between gender.

**Conclusions:**

In this study, 132 neonates were initially screened, of which 117 met the inclusion criteria and were finally included. There were 69 male and 48 female neonates. The indicators α, a, b, and c showed no statistical differences between male and female neonates; therefore, we combined the results to obtain the normal reference value: angle α: 86.34 ± 8.58°; distance a: 63.63 ± 6.83 mm; distance b: 31.99 ± 3.70 mm; distance c: 2.28 ± 1.04 mm.

## Introduction

The mandible is arch-shaped and located in the lower part of the face. It forms the front and side walls of the mouth. It is divided into the mandibular body and the mandibular ramus, with the bilateral mandibular body confluence at the mandibular median joint. There is a protrusion on the upper side of the mandibular ramus and a curved concave edge on the lower side, that is, the mandibular notch. The mandibular notch terminates forward on the other bony prominence called the condyle. The intersection of the posterior margin of the mandibular ramus and the lower edge of the mandibular body is the angle of the mandible. The mandible is a component of the temporomandibular joint, which is the only movable joint of the maxillofacial region and the only joint of the human body (1).

The diseases associated with the mandible include mandibular angle hypertrophy (2), mandibular defects (from osteomyelitis of the jaw, radiation-induced bone disease, maxillofacial tumors, and cysts), mandibular fractures, and congenital mandibular dysplasia (e.g., Pierre Robin sequence sign, Treacher Collins syndrome, first sacral arch syndrome, etc). The main treatment for these diseases is surgical intervention to appropriately restore the normal shape of the mandible. How to define and measure the normal shape of the mandible are research hotspots worldwide. Norris (3) measured the cranial bones of 53 infants at the museum. The results showed that the height of the mandibular ramus was well correlated with the age of infant death within 6 months. This study provided a reference range for the normal value of the mandible at different ages. Chung (4) included the measurements in the Norris study as a reference range for normal values in a case-control study of the Pierre Robin sequence and Treacher Collins syndrome (5) and measured the mandible of 50 girls aged 3–16 years by x-ray in 1966. The measurement included the length of the mandibular ramus, the length of the mandible, the total length of the mandible, and the bilateral mandibular corner spacing and double side spacing. Studies have shown that the depth of jaw growth is greater than the width, which is greater than the length. In 1967, they used the same indicators to measure the mandible of boys aged 3–16 years in a case-control study. However, there is no measurement data for newborns and neonates.

Mandible disease can occur at all ages and can be diagnosed and treated at all ages. Some congenital mandibular dysplasia, such as the Pierre Robin sequence, often leads to severe breathing and feeding difficulties in children (6) because of the short mandibular retraction, tongue drop, and other symptoms (7), and many children need surgery during the neonatal period. Treatment of the disease mainly involves bilateral mandibular distraction osteogenesis (8). Therefore, we conducted this study to create a reference range of the normal values of the mandibular index by measurement for the diagnosis of the Pierre Robin sequence, development of the surgical plan, and the pull setting after placement of the extender.

## Materials and methods

**Figure 1:**
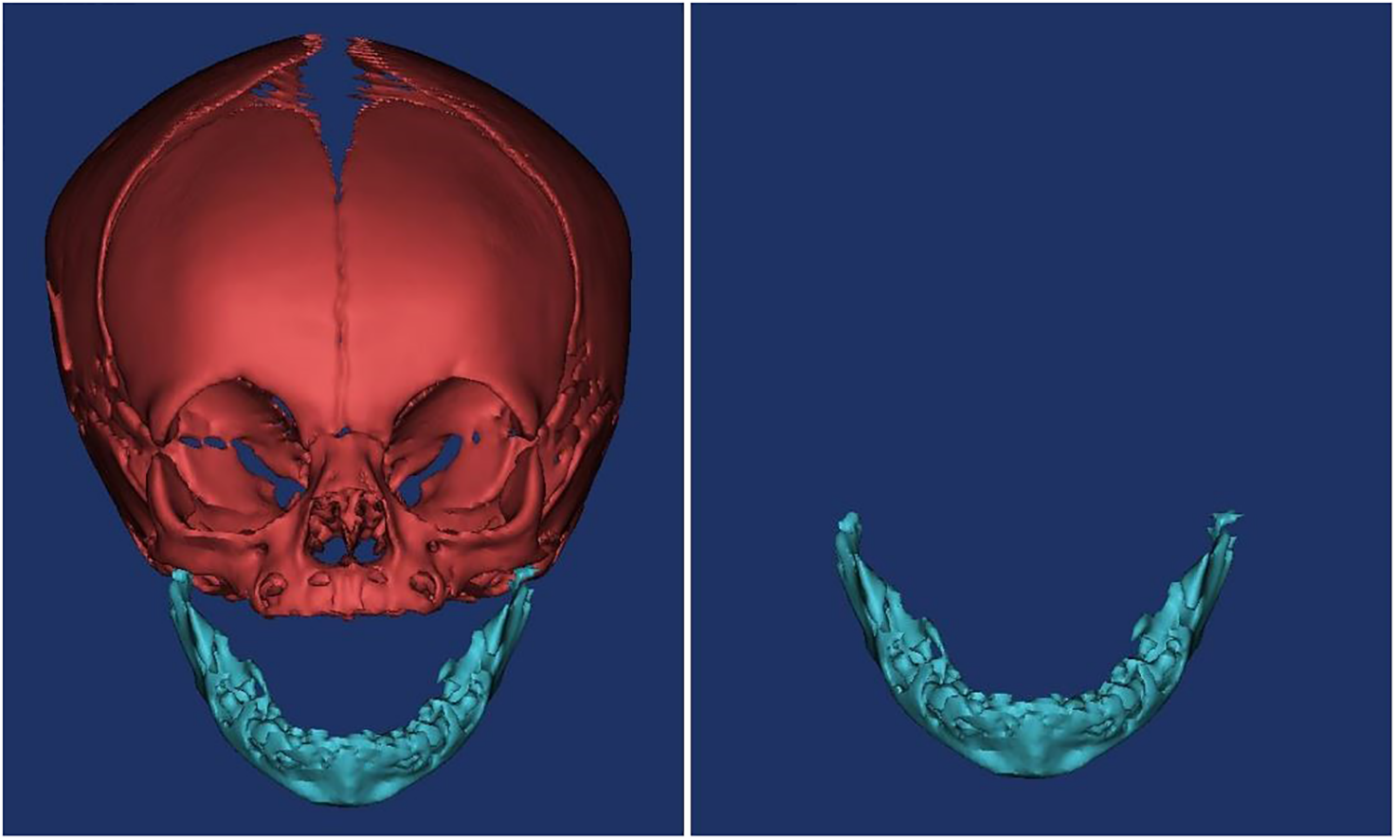
The 3D model.

We performed a retrospective analysis at the Children's Hospital of Nanjing Medical University. Neonates who underwent craniofacial skeleton CT examination between January 2013 and January 2019 were included. Inclusion criteria were as follows: age between 0 and 28 days, full-term infants, CT scans were performed due to various reasons such as craniofacial trauma, tumors, and other factors, CT data showing complete visualization of the maxilla and mandible, absence of mandibular deformities, and no craniofacial abnormalities. Exclusion criteria were the presence of other severe deformities. A total of 132 neonates were initially screened, of which 117 met the inclusion criteria and were finally included. There were 69 male and 48 female neonates. We stored all CT images in DICOM format, imported them into Mimics, and acquired 3D surface reconstructions of the skull (Figure 1). The models were used to evaluate the morphology of the mandible.

**Figure 2:**
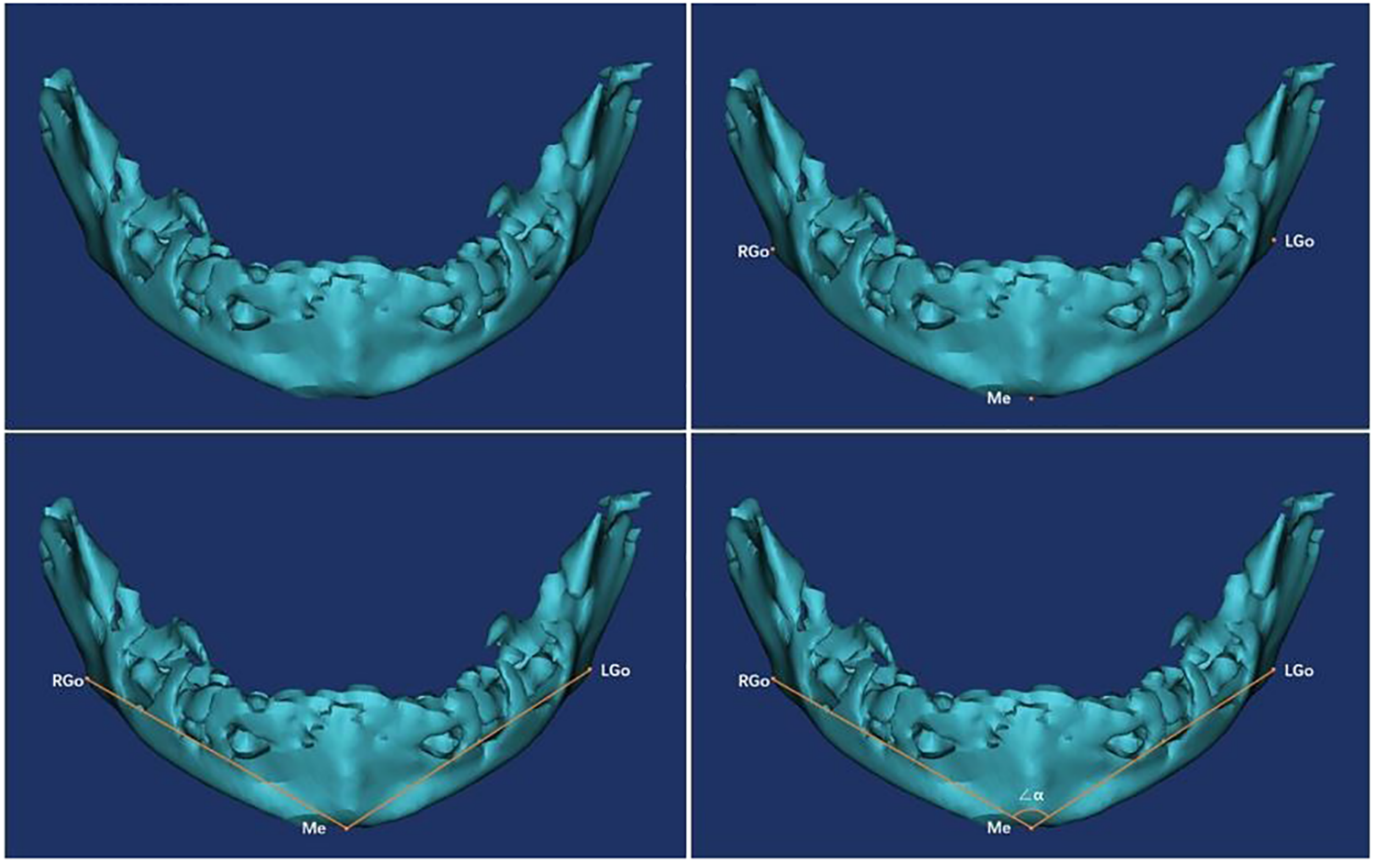
Measurement of the angle α: the 3D model was observed to find the left and right gonions (LGo and RGo) and the menton (Me), and the angle measurement tool of the software was used to appoint Me as the apex, and the points LGo, Me, and RGo were connected as angle α.

**Figure 3:**
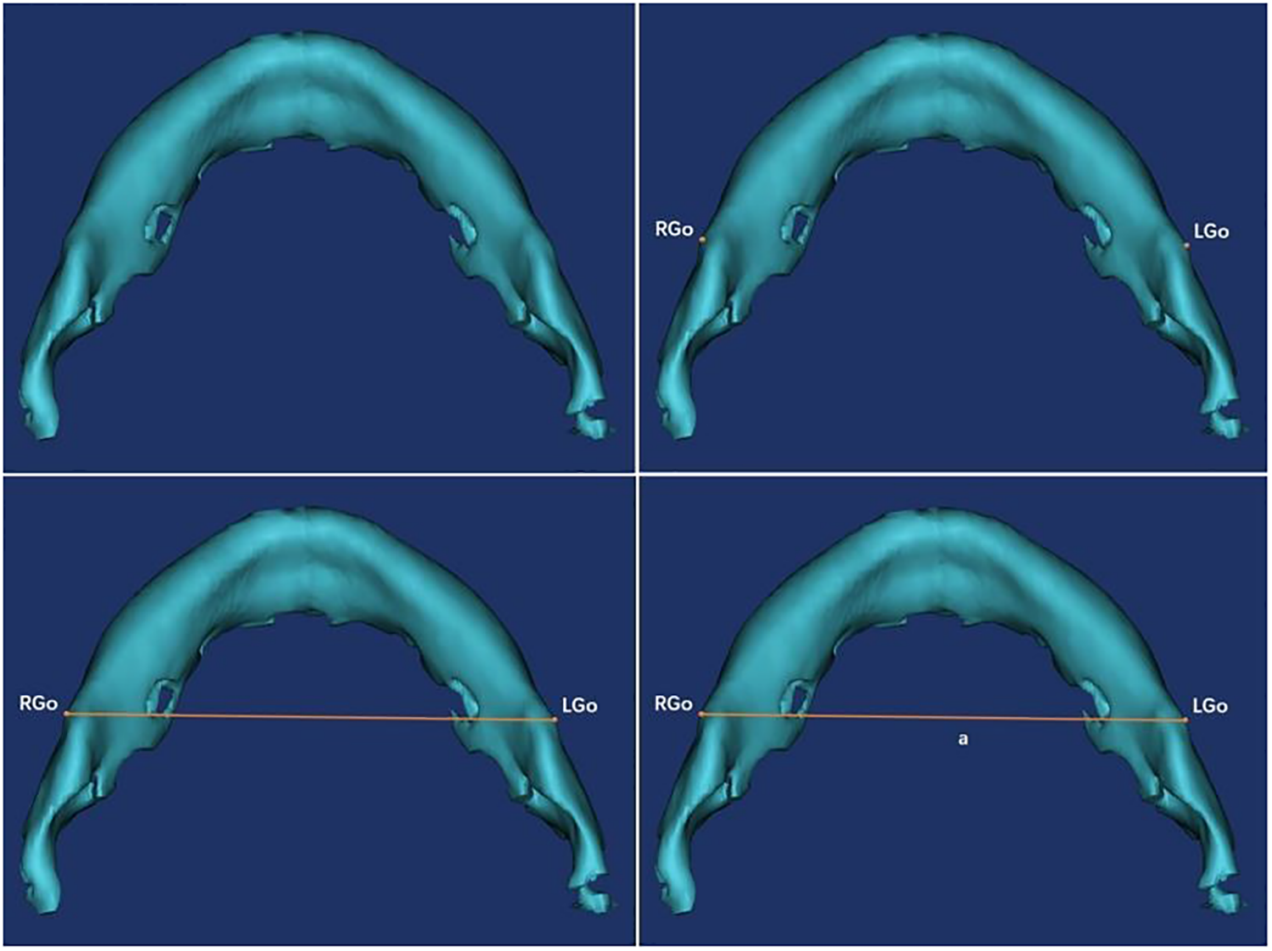
Measurement of the distance between the left and right gonions: the distance measurement tool of the software was used to measure the distance between the bilateral gonions as a.

**Figure 4:**
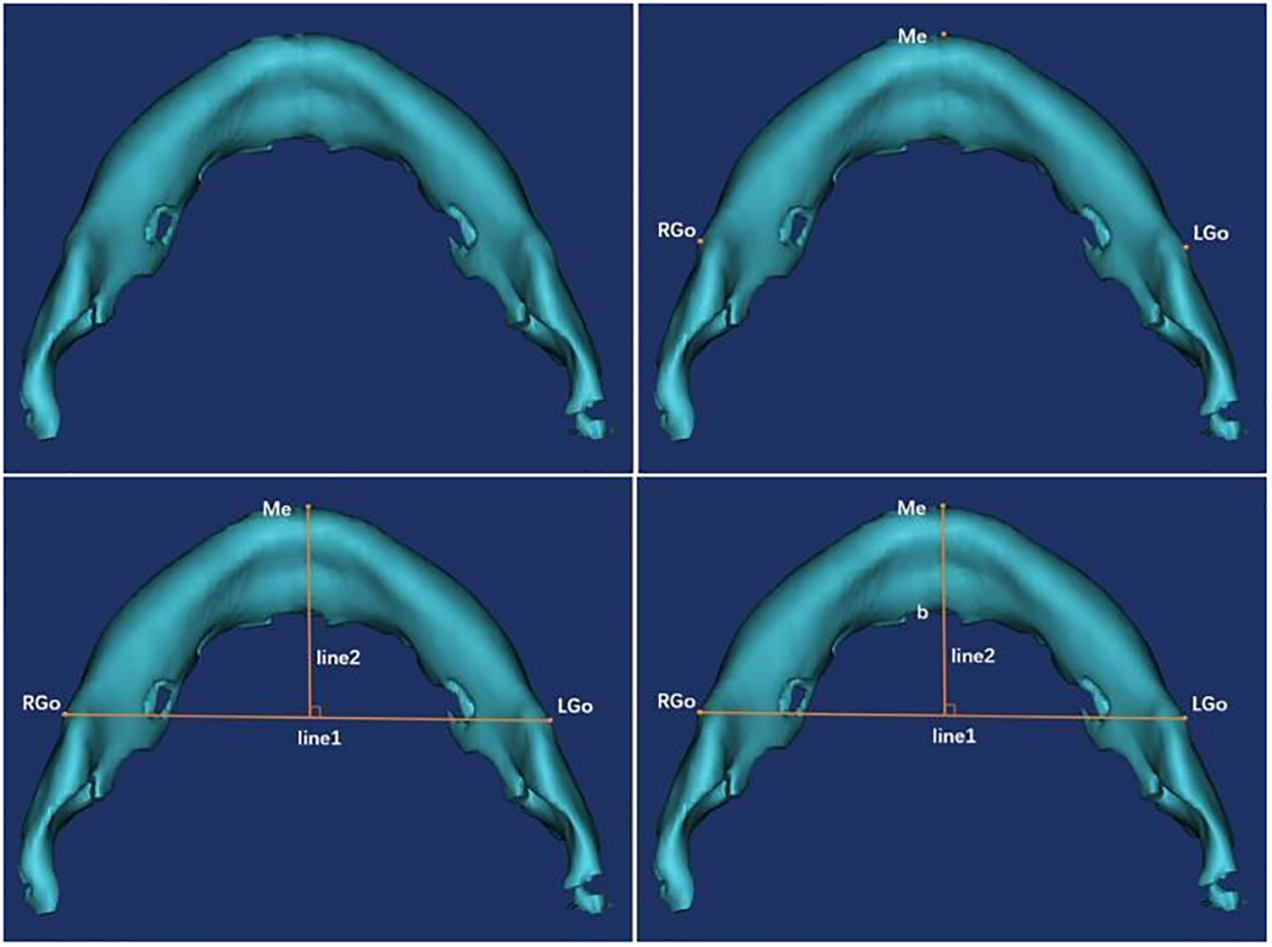
Measurement of the distance from Me to the line between LGo and RGo: the LGo and RGo were connected as a line on the 3D model, and then the distance between Me and the line was measured as b.

**Figure 5:**
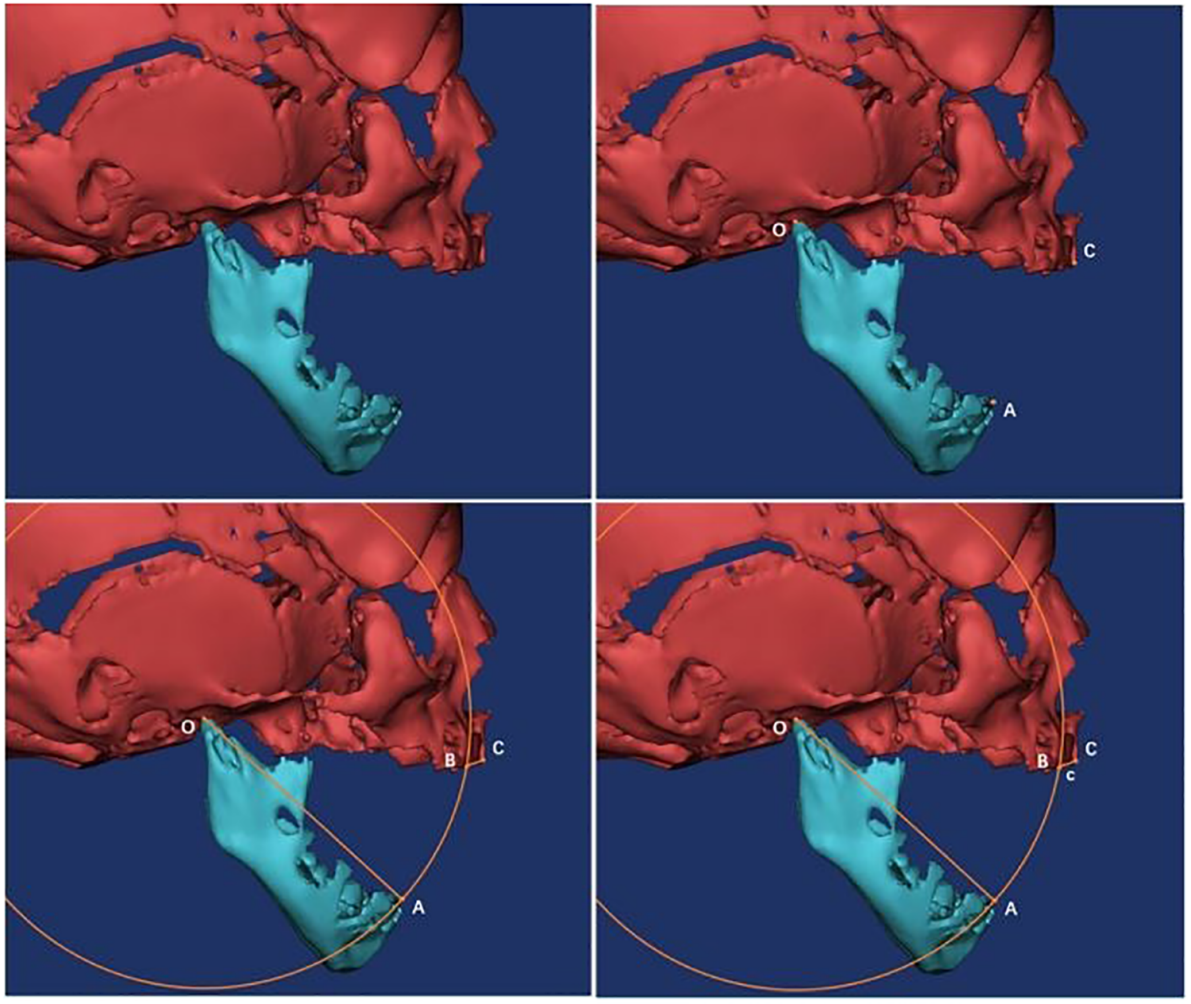
Measurement of the distance between the upper and lower jaw: the median sagittal view was found, and the distance c between the foremost point of the upper jaw and the foremost point of the lower jaw was measured.

Specific measurement items were as follows: ① Measurement of the angle α: We imported the CT data of the neonate into the software and reconstructed a 3D model. Then we observed the 3D model to find the left and right gonions (LGo and RGo) and the Menton (Me) and used the angle measurement tool of the software to appoint Me as the apex, and we connected points LGo, Me, and RGo as angle α (Figure 2). ② Measurement of the distance between the left and right gonions: The distance measurement tool of the software was used to measure the distance between the bilateral gonions as a (Figure 3). ③ Measurement of the distance from the Me to the line between LGo and RGo: The LGo and RGo were connected as a line in the 3D model, and then the distance between Me and the line was measured as b (Figure 4). ④ Measurement of the distance between the upper and lower jaw: The median sagittal view was found and the distance c between the foremost point of the upper jaw and the foremost point of the lower jaw was measured (Figure 5). We imported the measurement results into the SPSS software for statistical analysis.

## Results

Angle α: 86.34 ± 8.58°. The distance between the left and right gonions: 63.63 ± 6.83 mm. The distance b from the underarm point to the mandibular angle on both sides: 31.99 ± 3.70 mm. The distance c between the upper and lower jaws: 2.28 ± 1.04 mm (Table 1). Among all the above indicators, there was no statistical difference in gender.

**Table 1:** Angle α(X ± S,°); distance a(X ± S, mm); distance b(X ± S, mm); distance c(X ± S, mm).

Items	Sex	Numbers	X ± S	*P*	Result
Angle α	Male	69	86.65 ± 8.22	0.638	86.34 ± 8.58
	Female	48	85.89 ± 9.14		
Distance a	Male	69	63.51 ± 7.11	0.822	63.63 ± 6.83
	Female	48	63.80 ± 6.48		
Distance b	Male	69	32.03 ± 3.60	0.896	31.99 ± 3.70
	Female	48	31.94 ± 3.92		
Distance c	Male	69	2.34 ± 0.94	0.482	2.28 ± 1.04
	Female	48	2.20 ± 1.19		

## Discussion

There are several methods for measurement of the mandible, such as skull specimen measurements, *in vivo* measurements, x-ray imaging measurements, and 3D measurements. Skull specimen measurement involves the use of vernier calipers and other tools to measure the relevant data of skull specimens (9); *in vivo* measurement is the use of human anatomical landmarks directly measured on the human body (10); two-dimensional imaging measurement refers to the use of x-ray in combination with the skull locator to take a lateral slice of the median sagittal plane and measure the relevant data on the lateral slice. Commonly used 3D measurement techniques include 3D laser scanning technology, close-range stereo photography, structured light technology, 3D CT measurement technology, and CBCT measurement technology (11). 3D CT measurement technology and CBCT measurement technology are used for cranial and maxillofacial bone structures.

Skull specimens are directly measured with angle gauges, right angle gauges, vernier calipers, and other tools. The method is intuitive, simple, and economical (12). Therefore, it has been widely used at home and abroad, and extensive anatomical data from early human physique surveys were obtained by this method (13). However, the acquisition of skull specimens is difficult, and the age, gender, and other factors of the specimen are difficult to control. The measurement of skull specimens cannot meet the needs of the rapid development of modern medicine.

*In vivo* measurements are more common in epidemiological investigations, and anthropometric tools such as right angle gauges and triangular parallel gauges are used on the skin measurement points.

X-ray is a more accurate measurement of the hard tissue of the head and face. However, it acquires the image under the head locator, and the head scalar plane of the subject is adjusted to be parallel to the horizontal plane by the head aligner (14).

The 3D measurement in this study mainly refers to 3D CT reconstruction measurement technology, which exports the data obtained by CT measurement in DICOM format and then imports the corresponding 3D software to reconstruct the 3D model. Thereafter, the measurement tools of the software are used to conduct measurements on related data. Once the corresponding measurement point is determined in the image after the 3D reconstruction, the measurement data does not change with the rotation of the image. On the other hand, since the measurement is performed using the measurement tool of the software, the enlargement or reduction of the image does not affect the length index of the measurement. In addition, zoologists have already made CT 3D measurements in the measurement research of porcupines, Hamdani sheep, and Aksaray Malaklisi dogs and achieved satisfactory results (15–18). The method does not require the investigator to adjust the parallel plane of the flange plane to the horizontal plane with the aid of the head locator, which is particularly advantageous for the measurement of newborns.

For the treatment of the Pierre Robin sequence, the lighter can be used to adjust the position; the artificial position can be established by adjusting the position invalid (19). However, non-surgical treatment is only effective in some patients for relieving airway obstruction. Surgery is required for children if non-surgical treatment is not effective. The clinical application of lip and tongue adhesion can be traced back to 100 years ago and can be used to relieve respiratory airway obstruction in children (20). In addition to the high incidence of complications, the surgical procedure may have the possibility of recurrence of airway obstruction after the adhesion of the lip and tongue is removed. In addition, the operation does not significantly improve the shape and appearance of the child. Traction osteogenesis is performed directly on the mandible and is distracted into osteogenesis. The improvement of airway obstruction is increasingly obvious with the traction process (21). It also improves the shape and appearance of the patient and has been increasingly used in the clinic in recent years.

The angle α normal reference range is 86.34 ± 8.58°, and the bilateral mandibular angle spacing a normal reference range is 63.63 ± 6.83 mm. These two values can be used as a measure of the size of the mandible in the left and right directions. The distance from the kneeling point to the bilateral mandibular angle is normal. The reference range is 31.99 ± 3.70 mm, and the normal range of the upper and lower jaws is 2.28 ± 1.04 mm. These two values can be used as a measure of the size of the mandible in the anteroposterior direction. There were no gender differences in all the measurements, which may be related to the effects of neonatal sex hormones on bones.

We will continue to collect eligible CT scan data to expand the sample size and conduct measurements of the Pierre Robin sequence for mandibular distraction osteogenesis-related anatomy.

While expanding the sample size and increasing the measurement items, the collected data can be used to establish a standardized 3D model of the normal mandible of newborns in Jiangsu, China. This model can be used to design and develop traction equipment related to mandibular distraction osteogenesis, 3D simulation of traction equipment, and 3D finite element stress analysis of the traction process. Further research can integrate facial soft tissue data into a standardized 3D model, and the whole distraction osteogenesis process can be better simulated on the integrated model.

## Data Availability

The raw data supporting the conclusions of this article will be made available by the authors, without undue reservation.
